# Clinical severity and health-seeking behaviors of cases with hemorrhagic fever with renal syndrome: A comparative study across spatial clusters in Jiangxi Province, China

**DOI:** 10.1371/journal.pntd.0014746

**Published:** 2026-09-23

**Authors:** Guanghui Xia, Rui Gao, Huanhong Pan, Jun Zong, Zhili Zeng, Zifen Li, Chenwei Li, Shiyu Liao, Tong Luo, Guai Tang, Zijing Peng, Xiaoqing Liu, Shiwen Liu, Jianxiong Li, Tianchen Zhang

**Affiliations:** 1 Jiangxi Provincial Key Laboratory of Major Epidemics Prevention and Control/ Medical Leading Discipline of Jiangxi Province (Epidemiology), Jiangxi Provincial Center for Disease Control and Prevention, Nanchang, Jiangxi, China; 2 School of Public Health, Jiangxi Medical College, Nanchang University/ Jiangxi Provincial Key Laboratory of Disease Prevention and Public Health, Nanchang University, Nanchang, Jiangxi, China; Seoul National University College of Medicine, KOREA, REPUBLIC OF

## Abstract

**Background:**

Hemorrhagic fever with renal syndrome (HFRS) is a significant public health concern in Jiangxi Province, China, where both Hantaan (HTNV) and Seoul (SEOV) co-circulate. Previous studies have identified three distinct clusters of HFRS epidemic in Jiangxi Province, characterized by geographical separation and heterogeneous incidence levels. We hypothesized that clinical severity and healthcare-seeking behaviors may vary across these clusters, and thus conducted a retrospective study to examine this hypothesis.

**Methods:**

We retrospectively analyzed 4,614 HFRS cases (13-year period) from 63 counties. Cases were classified into four areas based on prior spatial study, representing distinct hilly and plain regions with different proximity to major water bodies and mountains. Data on HFRS cases were collected by county-level CDC staff using a nationally standardized case investigation questionnaire. Clinical severity and healthcare-seeking behaviors (hospital level, onset-to-visit interval) were compared.

**Results:**

HFRS cases in Cluster I exhibited the highest clinical severity (22.41% severe, 6.65% critical), while cases in Cluster II were strikingly mild (2.62% severe, 1.31% critical). Median onset-to-visit interval was longest in Cluster II (5 days) vs others (2–4 days). Hospital preference varied: Cluster I primarily used Class II hospitals (65.88%); Cluster II showed near-equal use of Class II (48.44%) and III (47.01%); Cluster III and non-clustered areas favored higher-level hospitals.

**Conclusion:**

Our findings confirmed that Jiangxi Province, though a mixed epidemic focus, exhibited distinct spatial heterogeneity in clinical severity, and healthcare-seeking behaviors. These findings underscore the need for regionally tailored strategies in HFRS prevention and control.

## Author summary

Hemorrhagic fever with renal syndrome (HFRS) is a serious infectious disease in China, with thousands of cases reported annually. Previous studies have shown that cases in Jiangxi Province tend to cluster in specific geographic areas. However, it remained unclear whether patients in these different areas experienced different disease outcomes or sought care in different ways. In this study, we analyzed data from more than 4,600 patients across 63 counties over 13 years. We found that disease severity and how quickly people sought care differed substantially between regions. Patients in Cluster I were much more likely to develop severe or critical illness, whereas cases in Cluster II were predominantly mild. We also identified significant inter-regional differences in healthcare-seeking behavior, including time to initial care and choice of healthcare facility. Cluster I patients predominantly utilized Class II hospitals, while those in other regions more often presented directly to Class III hospitals. These findings show that HFRS affects different parts of Jiangxi Province in different ways. This means that public health strategies—such as health education campaigns and hospital resource planning—should be tailored to the specific needs of each region, rather than using a one-size-fits-all approach.

## Introduction

Hemorrhagic fever with renal syndrome (HFRS) is a rodent-borne zoonotic disease caused by hantavirus (HVs) infection [1,2]. Humans are mainly infected through being bitten by infected host animals, inhaling or directly contacting their excretions and contaminants [3]. The etiology of HFRS exhibits geographic variation: it is primarily caused by Hantaan (HTNV) and Seoul (SEOV) viruses in Asia, and by Puumala (PUUV) and Dobrava-Belgrade (DOBV) viruses in Europe, where the infection often presents as the milder nephropathia epidemica (NE) [4]. China bears the highest global burden of HFRS, accounting for over 90% of reported cases worldwide [5,6]. During the period from 2004 to 2019, a total of 209,209 cases of HFRS were reported, with a case fatality rate(CFR) of 0.89% [7]. In recent years, there has been a coexistence of multiple HVs gene branches [8], an expanding epidemic area, an increasing number of mixed epidemic areas, and a rapid increase in the infection risk among the elderly population [9,10].The domestic HFRS epidemic sources are mainly distributed in plains and hilly areas with an altitude of less than 500 meters, areas with abundant water in temperate and subtropical monsoon climate zones, and regions where wet animals are geographically distributed [11].

Jiangxi is a high-incidence area for HFRS in China and is characterized by the co-circulation of both HTNV and SEOV [12,13]. During 2005 and 2024, almost 10,000 HFRS cases were reported, with a CFR of 1.1%. Furthermore, the disease was reported in over 90% of all counties and districts. Previous studies have found that the incidence of HFRS in Jiangxi Province shows spatial clustering [14]. HFRS cases are concentrated in the Chiu-ling Mountains and then spread to the Wuyi Mountain hills over time, and three clusters were detected [12]. The Cluster I, situated in the northwestern region between the Chiu-ling Mountains and the western side of Poyang Lake, represents a historical high-incidence area (average annual rate: 12.10 per 100,000) [12]. In contrast, The Clusters II and III, located to the east and south of Poyang Lake and within the Wuyi Mountains region, exhibited progressively lower incidence rates of 4.20 and 1.80 per 100,000, respectively [12]. HTNV strains in Jiangxi were in one independent branch, while the SEOV strains in Jiangxi were relatively more diverse [13]. Laboratory evidence from one county in Cluster I indicated the exclusive presence of HTNV and the absence of SEOV [15].

The distinctive spatial patterning of HFRS in Jiangxi Province may be related to two primary factors. First, natural barriers, including mountain ranges and Poyang Lake, likely restrict host movement and viral gene flow among the different clusters [16,17]. Second, variations in altitude contribute to divergent ecological niches for reservoir rodent species [18]. Building on the observed correlation between spatial distribution and incidence rates, we hypothesize that: (1) in high-incidence areas, residents tended to have shorter onset-to-visit intervals, and local health facilities had more extensive experience in the diagnosis and treatment of the disease; and (2) influenced by this spatial and environmental heterogeneity, the prevalence of SEOV and HTNV, as well as the host carriage rates, may vary, with potential implications for differences in disease severity across populations. To test these hypotheses, we conducted a retrospective study investigating the clinical severity and the healthcare-seeking behaviors of HFRS cases.

## Materials and methods

### Ethics statement

This study was based on data collected through routine epidemiological investigations conducted by county-level CDC professionals as part of the statutory notifiable infectious disease surveillance system in Jiangxi Province, China. The study was reviewed and approved by the Ethics Review Committee of Jiangxi Provincial Center for Disease Control and Prevention (approval number: Gan CDC Ke Lun Shen (2025) No. 04). The requirement for informed consent was waived because the data were derived from routine statutory surveillance and the study posed no additional risk to subjects, in accordance with Chinese regulations governing the use of statutory communicable disease surveillance data for public health purposes.

### Study area

Jiangxi is located in south eastern China (24°29′14″–30°04′44″N, 113°34′36″–118°28′58″E), a humid subtropical climate area that includes 11 cities and 100 counties. It is surrounded by mountains on the east, south and west sides, with extensive hills in the inner area and a vast plain in the north and central parts [19]. The southern part of the province is hills with ranges and valleys interlaced; while the central and northern parts have a relatively flat terrain with lower altitudes. The entire province extends from south to north, tilting towards Poyang Lake, which forms a huge basin, with an open area to the north [19]. Previous spatial analyses [12] have identified three distinct clusters of HFRS cases in Jiangxi Province (S1 Text). The Cluster I is located on the west side of the northern hilly area and includes some plain counties adjacent to rivers and lakes. The Cluster II areas is located on the east side of the northern hilly area. The Cluster III is located on the south side of the northern hilly area, and also includes some plain counties adjacent to rivers and lakes. The Cluster II and Cluster III are both close to the Wuyi Mountains, and the Cluster III borders the Cluster I.

### Epidemiological investigations

In accordance with the *National Surveillance Program for Hemorrhagic Fever with Renal Syndrome*, epidemiological investigations were promptly initiated by trained professionals from the Centers for Disease Control and Prevention after hospital notification of HFRS cases — including both clinically diagnosed and laboratory-confirmed cases as defined by the WS278–2008 criteria (S2 Text) —using the nationally standardized HFRS case investigation questionnaire to obtain field information (S3 Text) on general patient characteristics, laboratory diagnosis situation, clinical severity, and healthcare-seeking behaviors with supplementary telephone interviews conducted when necessary. Personal and healthcare-access details were provided by patients themselves or by family members when the patient was unable to communicate—for example, due to death, severe illness, or young age. Clinical information was obtained by consulting treating physicians and reviewing medical records. Based on the clinical characteristics, HFRS severity was divided into four clinical types (S4 Text): mild (renal injury without hypotension or oliguria), moderate (uremia with acute renal failure and oliguria), severe (hypotension with severe uremia and oliguria), and critical (refractory shock, organ failure, or severe renal failure). Severity classification was determined by attending physicians for surviving patients based on the above standardized criteria at the time of admission.

### Study participants

A total of 7,172 cases were reported during the study period from 2011 to 2023. As the national surveillance program did not mandate case-based investigation for all counties, 2,374(33.10%) cases lacked standardized epidemiological data. Among the remaining 4,798(66.90%) cases included for initial review, we performed detailed verification and excluded those with logical error (visit date later than onset date; age inconsistent with reported occupation) or lack of key information (age, gender, current address, visit date and onset date). Consequently, 4,614(64.33%, 4,614/7,172) cases were included in the final analysis, with 2,320 cases in Cluster I, 1,120 cases in Cluster II, 542 cases in Cluster III, and 632 cases in non-clustered areas (Fig 1). The included cases (4,614) were largely comparable to all reported cases (7,172) in terms of gender, age, and main occupational composition, and their epidemic trends were also highly similar (S1 Fig). Furthermore, the included (4,614) and excluded (2,558) cases exhibited similar age and gender distributions, suggesting that selection bias was limited (S2 Fig). Collectively, these findings indicate that the analytic sample was highly representative.

**Fig 1:**
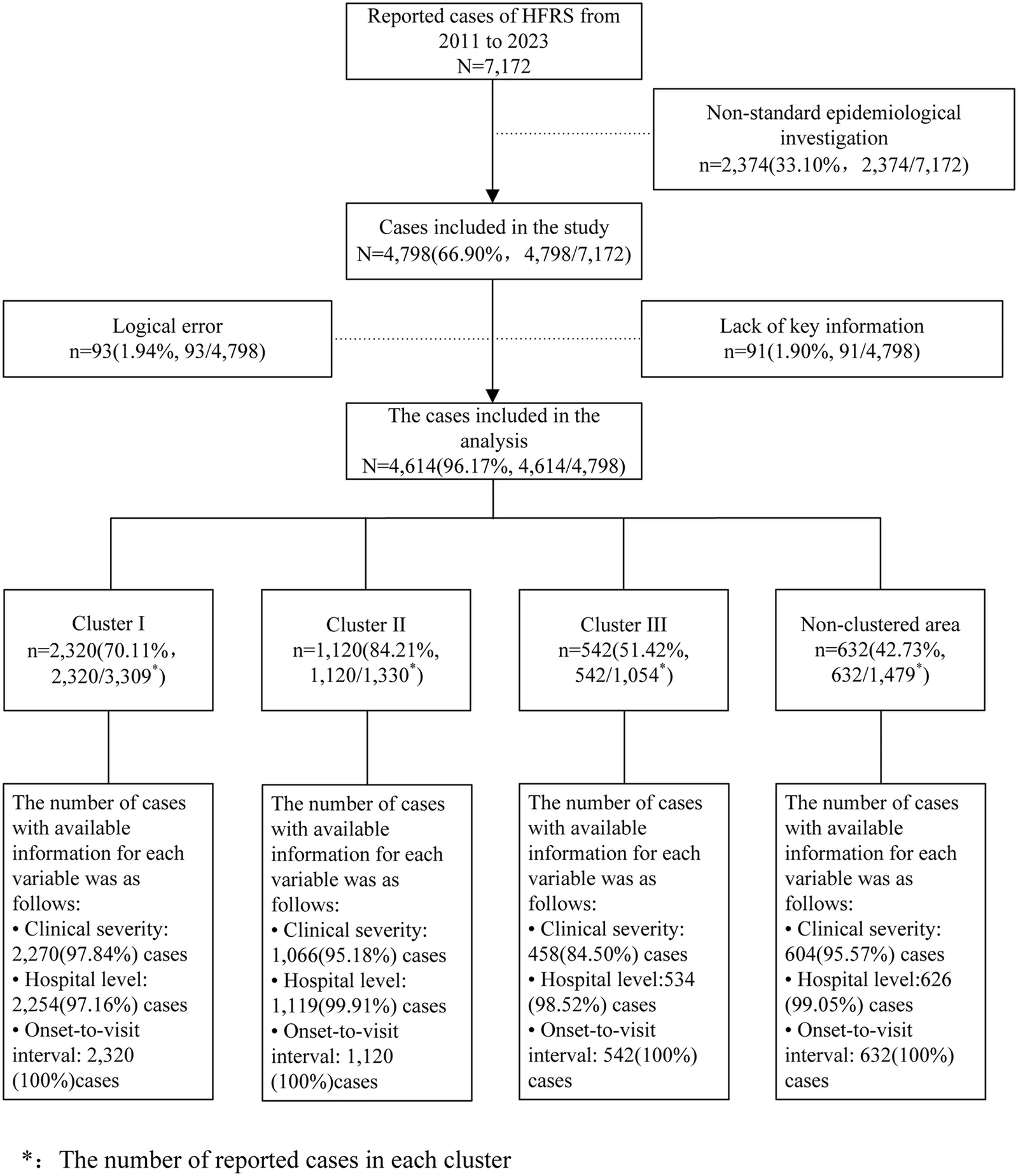
Flowchart for inclusion of HFRS cases in the analysis.

### Statistical analysis

Differences across clusters were compared using the Kruskal-Wallis H test for continuous variables, and the Chi-square or Fisher’s exact test for categorical variables. Post-hoc pairwise comparisons were performed with Bonferroni correction when global tests were significant. To adjust for potential confounders, two separate multivariable logistic regression models were constructed: one for clinical severity (mild/moderate vs. severe/critical) and one for hospital level (Class II vs. Class III/III+), with cluster, age, sex, occupation, and year of onset as covariates. Adjusted odds ratios (aORs) with 95% CIs were reported. All statistical analyses were conducted using R software (version 4.3.0), and a *P* < 0.05 was considered to be significant.

## Results

### Patients’ general characteristics

From 2011 to 2023, a total of 7,172 cases were reported in Jiangxi Province, corresponding to a cumulative incidence rate of 1.64 per 100,000 population. The annual reported incidence rate showed an overall downward trend (S3 Fig), declining with fluctuations from 1.24 per 100,000 in 2011 to 0.66 per 100,000 in 2023. Substantial variation in cumulative incidence was observed across clusters: the rates were 7.50, 3.27, and 1.46 per 100,000 for Clusters I, II and III, respectively, compared with 0.34 per 100,000 in the non‑clustered areas. Among the 4,614 analyzed cases, the male-to-female ratio was 2.09:1 (3,123 vs. 1,491), which did not differ significantly across the four areas. The mean age was 49 ± 18 years. The proportion of cases in the 0–20 age group was significantly higher in Cluster I (14.44%) than in other areas (4.59% ~ 6.46%), whereas the opposite trend was observed for the 41–60 age group (37.20% vs. 46.25% ~ 49.45%). The proportion of students in Cluster I was significantly higher than those other areas, while the proportion of farmers in non-cluster areas was significantly lower than other areas (Table 1).

**Table 1:** The characteristics of HFRS cases by clusters.

Characteristics	Cluster I	Cluster II	Cluster III	Non-clustered area	χ^2^/*H*	*P*
Gender					4.43	0.218
Male	1,550(66.81%)	754(67.32%)	387(71.40%)	432(68.35%)		
Female	770(33.19%)	366(32.68%)	155(28.60%)	200(31.65%)		
Gender ratio	2.01	2.06	2.50	2.16		
Age(years)					151.10	< 0.001
0–10	127(5.47%)	11(0.98%)	17(3.14%)	7(1.11%)	58.535	< 0.001
11–20	208(8.97%)	53(4.73%)	18(3.32%)	22(3.48%)	47.291	< 0.001
21–30	227(9.78%)	87(7.77%)	55(10.15%)	61(9.65%)	4.307	0.230
31–40	307(13.23%)	154(13.75%)	71(13.1%)	100(15.82%)	3.005	0.391
41–50	448(19.31%)	244(21.79%)	135(24.91%)	147(23.26%)	11.266	0.010
51–60	415(17.89%)	274(24.46%)	133(24.54%)	146(23.1%)	27.438	<0.001
61–70	376(16.21%)	204(18.21%)	79(14.58%)	100(15.82%)	4.203	0.240
71–80	171(7.37%)	82(7.32%)	32(5.9%)	40(6.33%)	2.079	0.556
≥ 81	41(1.77%)	11(0.98%)	2(0.37%)	9(1.42%)	8.015	0.046
Occupation					217.31	< 0.001
Farmers	1633(70.39%)	841(75.09%)	419(77.31%)	379(59.97%)	57.225	< 0.001
Students	252(10.86%)	44(3.93%)	28(5.17%)	24(3.80%)	74.738	< 0.001
Workers	79(3.41%)	19(1.7%)	16(2.95%)	36(5.70%)	20.949	< 0.001
Housework/unemployment	114(4.91%)	89(7.95%)	28(5.17%)	76(12.03%)	45.614	< 0.001
Nursery children	22(0.95%)	2(0.18%)	1(0.18%)	1(0.16%)		0.007
Scattered children	17(0.73%)	1(0.09%)	1(0.18%)	0(0%)		0.006
Others	203(8.75%)	124(11.07%)	49(9.04%)	116(18.35%)	49.854	< 0.001
Total cases	2,320(50.25%)	1,120(24.26%)	542(11.74%)	632(13.69%)		
Proportion of severe/critical (%)	29.06% (660/2271^*^)	3.93% (42/1068^*^)	22.00% (101/459^*^)	14.21% (86/605^*^)	306.567	< 0.001
CFR	1.94% (44/2,271^*^)	0.47% (5/1,068^*^)	2.18% (10/459^*^)	3.31% (20/605^*^)	19.147	< 0.001
Median onset-to-visit interval (IQR)	3(1-4)	5(2-10)	2(1-5)	4(2-8)	292.540	< 0.001
Proportion attending Class III/III+ hospitals (%)	33.81% (762/2,254^#^)	50.31% (563/1,119^#^)	75.28% (402/534^#^)	72.36% (453/626^#^)	485.409	< 0.001

* Number of cases with clinical severity classification in each cluster; # Number of cases with hospital level in each cluster.

### Distribution of clinical severity by clusters

Among all the analyzed cases, 4,403(95.43%, 4,403/4,614) cases had clinical severity information. The proportions of mild, moderate, severe and critical cases were 49.15%, 30.66%, 15.17% and 5.02% respectively. A higher proportion of mild cases was observed in Cluster II and non-clustered areas, whereas severe and critical cases were more common in Cluster I and Cluster III. Furthermore, the proportion of critical cases in Cluster I was higher than Cluster III (Fig 2 and S1 Table).

**Fig 2:**
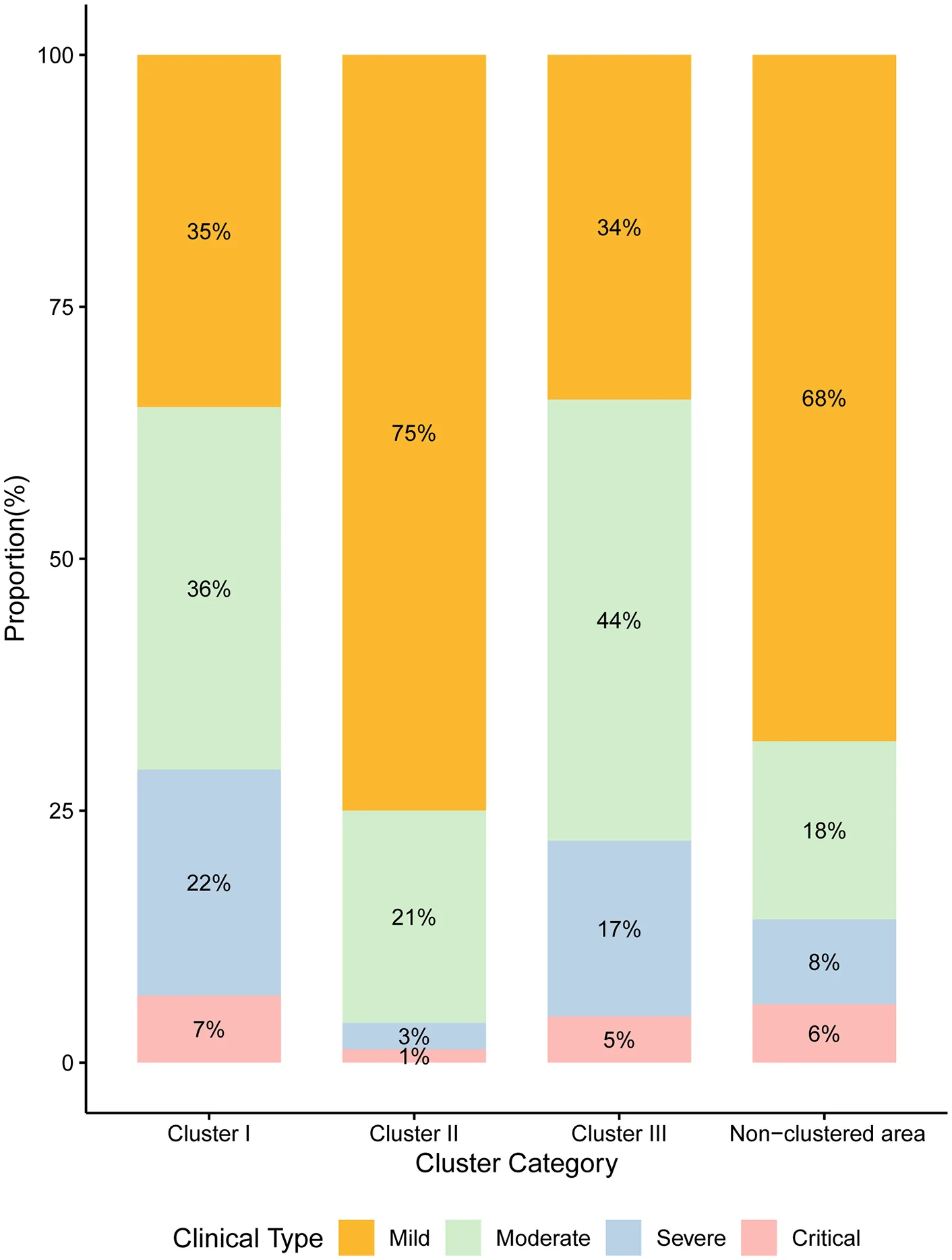
The distribution of clinical type of HFRS cases by clusters.

A total of 79 fatal cases were recorded, resulting in an overall CFR of 1.79% (79/4,403). The CFRs were similar in Cluster I (1.94%) and Cluster III (2.18%), which were higher than that in Cluster II (0.47%). Notably, the highest CFR (3.31%) was observed in non-clustered areas (Table 1).

### The healthcare-seeking behaviors

Four hospitals in Nanchang, the provincial capital, were selected as top-tier hospitals (Class III+): Jiangxi Provincial People’s Hospital, the First and Second Affiliated Hospitals of Nanchang University, and Nanchang Ninth Hospital. The first three were the leading general hospitals in Jiangxi, while the last was the province’s premier infectious disease hospital. As regional medical centers, they attracted patients from across the province.

Of the 4,533 cases with a documented hospital visit, 0.55% sought care at Class I hospitals, 51.36% at Class II, 31.41% at Class III, and 16.68% at Class III+ hospitals. A clear descending trend in patient proportion was observed from Class II to Class III+ hospitals. Stratified by cluster, we observed that this phenomenon was evident in Cluster I and II but not in Cluster III or non-clustered areas. In contrast, non-clustered areas exhibited the opposite pattern, with the highest proportion of patients seeking care at Class III+ hospitals (38.50%) (S2 Table).

HFRS typically progresses through five phases: febrile, hypotensive shock, oliguric, polyuric, and convalescent. The first four generally occur within one month of onset [20], and to our knowledge, no cases in the literature have sought initial care >30 days after onset. Given this, the 22 cases in our analysis with an onset-to-visit interval exceeding 60 days were considered recording errors. After restricting the analysis to cases with an interval of ≤60 days, 4,592 cases were included. Cases in Cluster II had a significantly longer onset-to-visit interval (median:5 days [interquartile range (*IQR*) 2–10]) than Cluster I (3 days [*IQR* 1–4]), Cluster III (2 days [*IQR* 1–5]), and non-clustered areas (4 days [*IQR* 2–8]) (all *P* < 0.001). Notably, interval in non-clustered areas also exceeded those in Cluster I (*P* < 0.001) and III (*P* < 0.001) (S4 Fig). This pattern of longer interval in Cluster II and non-cluster areas remained consistent in all subgroups stratified by gender, age, and occupation (S5 Fig). Regarding temporal trends, the proportion of cases seeking care within one week of onset consistently exceeded 85% across most years in both Cluster I and Cluster III (Fig 3).

**Fig 3:**
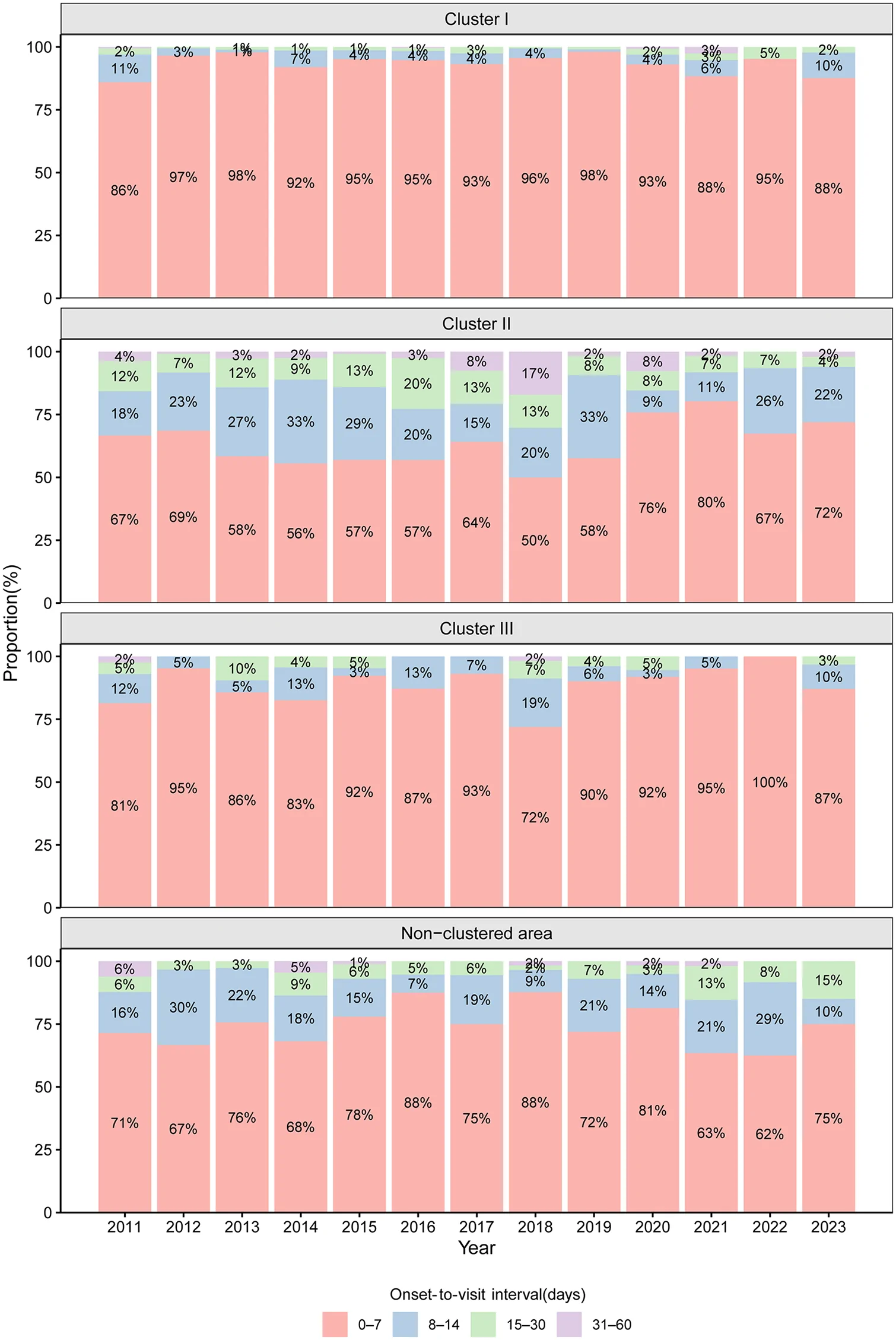
Temporal trends in onset-to-visit interval by clusters.

The median onset-to-visit interval was significantly longer in mild cases (4 days [*IQR*: 1–8]) than in moderate (3 days [*IQR*: 1–5]), severe (3 days [*IQR*: 1–4]), and critical cases (3 days [*IQR*: 2–5]) (all *P* < 0.001; S6 Fig). Significant differences were also observed across hospital levels (*P* < 0.001). Specifically, cases at Class III hospitals experienced the longest onset-to-visit interval (median:4 days [*IQR*: 2–8]), followed by those at Class II (3 days [*IQR*: 1–5]), Class III+ (2 days [*IQR*: 0–5]), and Class I (1 day [*IQR*: 0–4]) hospitals. Although the interval increased progressively from Class I to Class III, this trend did not reach statistical significance, potentially due to the limited number of cases visit to Class I hospitals (0.55%, 25/4511). Notably, the interval at Class III+ hospitals was shorter than that observed at Class II and Class III facilities (S7 Fig).

### Hospital level distribution of cases by clusters

Visits to Class I hospitals were uncommon overall, accounting for less than 0.4% of cases in most areas, with a slightly higher proportion observed in Cluster II (1.25%). Hospital preferences varied markedly across clusters. In Cluster I, the majority of cases sought care at Class II hospitals (65.88%), whereas a much smaller proportion attended Class III facilities (19.17%). Cluster II exhibited a nearly even distribution between Class II (48.44%) and Class III (47.01%) hospitals, while having the lowest rate of Class III+ visits (3.31%) among all groups. In contrast, the proportion of cases seeking care at Class III+ hospitals was substantially higher in Cluster III (27.72%) and non-clustered areas (38.50%). Overall, the combined proportion of patients attending Class III and Class III+ hospitals, in descending order, was: Cluster III (75.29%), non-clustered areas (72.37%), Cluster II (50.32%), and Cluster I (33.81%). (S8 Fig). Temporal trend analysis revealed that cases in Cluster I-III increasingly sought care at higher-tier hospitals over time (Fig 4).

**Fig 4:**
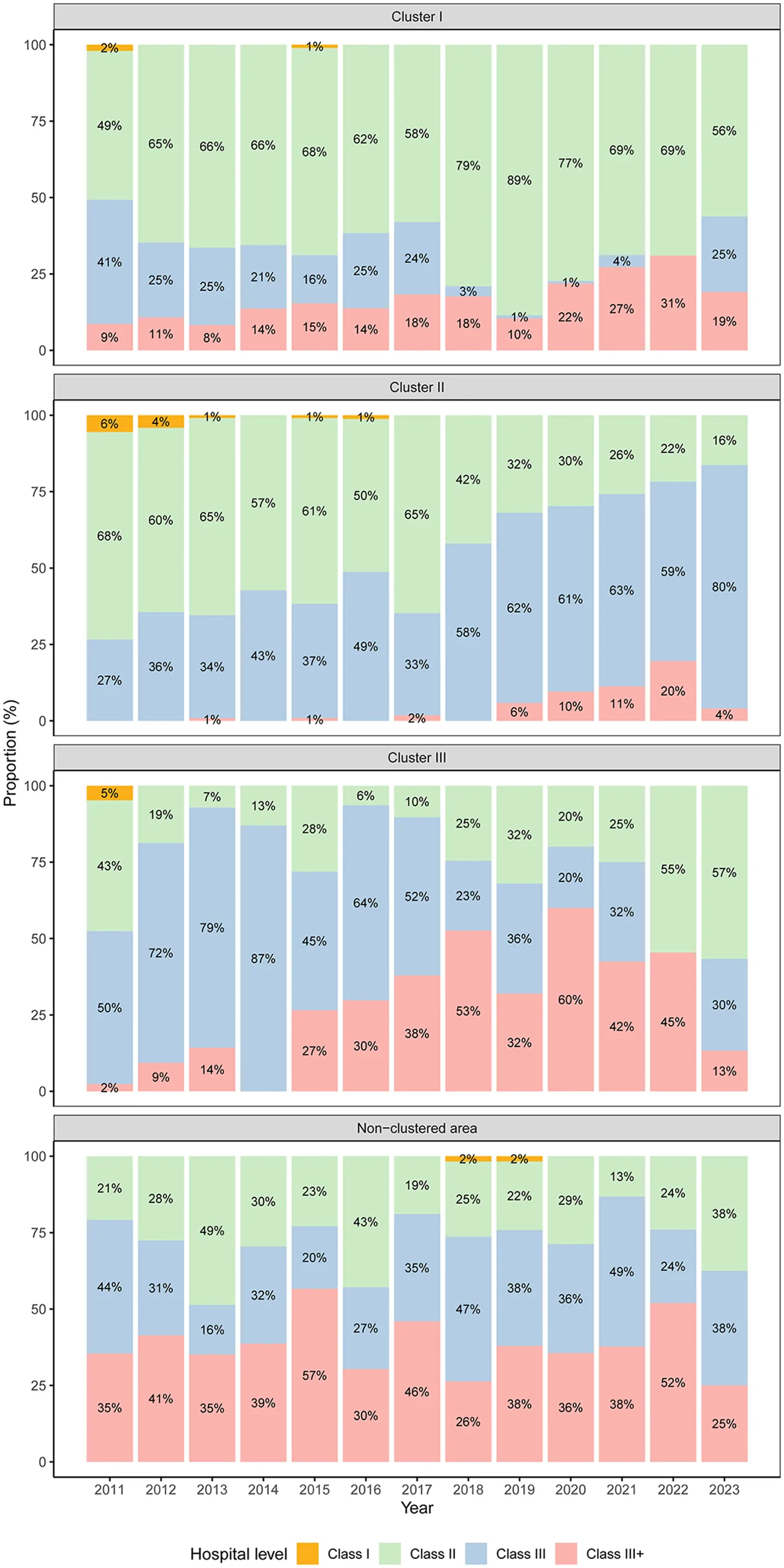
Temporal variation in hospital level distribution by clusters.

Subgroup analyses indicated that hospital level distribution was similar between genders within the same cluster (*P* = 0.07). Across all areas, the youngest age group (0–15 years) had the highest proportion of seeking care at Class III/III+ hospitals, with this proportion declining as age increased. Farmers consistently showed lower rates of seeking care at Class III/III+ hospitals compared with other occupations, except in Cluster III. In contrast, students exhibited a markedly higher propensity to utilize these higher-tier hospitals, with rates consistently exceeding the regional average—most notably in Cluster III, where 92.59% of students sought care at Class III/III+ facilities. (Fig 5)

**Fig 5:**
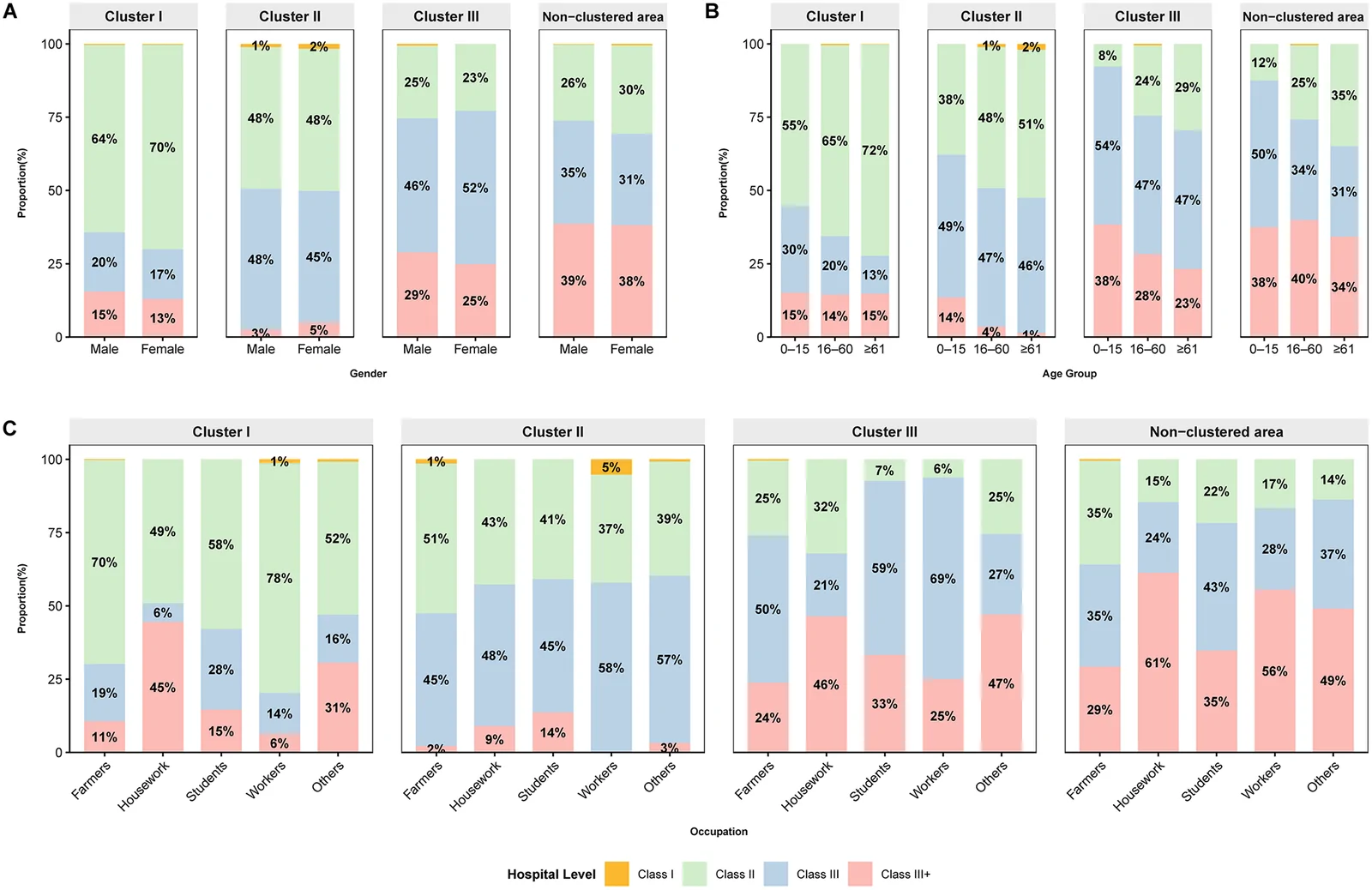
Comparison of hospital level distribution across clusters. **(A)** Stratified by gender; **(B)** Stratified by age; **(C)** Stratified by occupation.

The use of Class I hospitals was restricted to mild (0.56%) and moderate (0.75%) cases, with severe and critical cases being entirely absent. The proportion of visits to Class III+ hospitals increased with disease severity, rising from 12.48% in moderate cases to 14.01% in severe cases and 19.08% in critical cases. Although mild cases were the least symptomatic, their proportion of visits to Class III+ hospitals was significantly higher than that of moderate and severe cases and approached that of critical cases (S9 Fig). By cluster analysis, among all mild cases, the lowest proportion visiting Class III and Class III+ hospitals was observed in Cluster I (25.60%). In Cluster II, the highest proportion of visits to Class III and Class III+ hospitals occurred among mild cases (59.13%), while for other three categories of areas, the highest proportion was observed among severe or critical cases (S10 Fig).

### Laboratory confirmation rates by clinical severity and hospital level

We further examined laboratory confirmation rates by severity and hospital level (Table 2). Across all severity categories, rates varied substantially across clusters, with Cluster II consistently showing the lowest rates (33.03% for moderate to 56.0% for severe) and Cluster III the highest in severe (77.33%) and critical (75.00%) categories. Notably, confirmation rates did not show a clear gradient with increasing severity in any cluster. Across all clusters, laboratory confirmation rates also varied substantially by hospital level. In Non‑clustered areas, the highest rate was observed in Class II hospitals (82.39%), followed by Class III+ (70.62%) and Class III (69.54%), though Class I was based on a single case (100.0%, n = 1). In Cluster II, rates were comparable between Class II (43.84%) and Class III+ (41.94%), both markedly higher than Class III (25.30%). Notably, in Cluster I and Cluster III, Class III+ hospitals demonstrated the highest rates (79.82% and 82.27%, respectively).

**Table 2:** Laboratory confirmation rates by clinical severity and hospital level.

Index	Cluster I	Cluster II	Cluster III	Non-clustered area
Clinical type				
Mild	71.21% (512/719)	34.52% (271/785)	49.01% (74/151)	74.06% (237/320)
Moderate	69.82% (546/782)	33.03% (73/221)	38.97% (76/195)	81.11% (73/90)
Severe	69.43% (343/494)	56.00% (14/25)	77.33% (58/75)	70.59% (24/34)
Critical	63.95% (94/147)	53.85% (7/13)	75.00% (15/20)	61.54% (16/26)
Hospital level				
Class I	40.00% (2/5)	15.38% (2/13)	0.00% (0/2)	100.00% (1/1)
Class II	70.84% (1035/1461)	43.84% (235/536)	32.79% (40/122)	82.39% (117/142)
Class III	59.47% (245/412)	25.30% (127/502)	40.96% (77/188)	69.54% (105/151)
Class III+	79.82% (178/223)	41.94% (13/31)	82.27% (116/141)	70.62% (125/177)

### Multivariable regression analyses for clinical severity and hospital level

Two separate multivariable logistic regression models were constructed, with clinical severity (mild/moderate vs. severe/critical) and hospital level (Class II vs. Class III/III+) as the dependent variables, respectively, adjusting for cluster, age, sex, occupation, and year (Table 3).

**Table 3:** Multivariable logistic regression analyses of clinical severity and hospital level.

Variable	Severe/critical (ref: mild/moderate) aOR (95% CI)	Class III/III+ (ref: Class II) aOR (95% CI)
Cluster category (ref: Non-clustered area)		
Cluster I	2.20*** (1.71–2.86)	0.20*** (0.17–0.25)
Cluster II	0.24*** (0.16–0.35)	0.42*** (0.34–0.53)
Cluster III	1.58** (1.14–2.20)	1.29 (0.98–1.68)
Age (per 1-year increase)	1.00 (1.00–1.01)	0.99*** (0.98–0.99)
Gender (ref: Female)		
Male	1.22* (1.02–1.45)	1.19* (1.04–1.37)
Occupation (ref: Farmers)		
Students	1.08 (0.76–1.53)	1.06 (0.80–1.42)
Workers	0.75 (0.46–1.19)	0.94 (0.66–1.35)
Housework	0.73 (0.50–1.05)	2.01*** (1.54–2.61)
Others	1.25 (0.97–1.60)	1.75*** (1.43–2.16)
Year of onset (per 1-year increase)	0.94*** (0.92–0.96)	1.04*** (1.02–1.06)

* *p* < 0.05, ** *p* < 0.01, *** *p* < 0.001.

For severity, patients in Cluster I and Cluster III exhibited a significantly higher risk of severe/critical illness, while those in Cluster II showed a markedly lower risk, compared with non-clustered cases. Male sex was associated with 22% increased odds of severe disease relative to females. Each 1‑year increase in the year of onset was significantly associated with 6% reduction in the odds of severe outcomes. Age and all occupation subcategories showed no statistically significant associations with severity in the adjusted model.

For hospital level, patients in Cluster I and Cluster II were significantly less likely to be treated in Class III/III+ hospitals compared with non-clustered patients, with risk reductions of 80% and 58%, respectively. Cluster III showed a positive trend toward Class III/III+ care, though the association did not reach statistical significance. Older age was weakly but significantly associated with a lower probability of Class III/III+ admission, whereas males had higher odds than females. Individuals engaged in housework and other occupations were significantly more likely to be treated at Class III/III+ hospitals than farmers. Each 1‑year increase in onset year was associated with 4% increased odds of tertiary hospital admission.

## Discussion

As far as we know, this study is the first to focus on the differences in HFRS cases among various clusters in Jiangxi Province. The HFRS cases were mainly concentrated in the Cluster I, accounting for more than half of the total reported cases in the province. The male-to-female ratio among HFRS cases in Jiangxi Province was 2.09:1, which was lower than those observed in neighboring Zhejiang (2.56:1) [21] and Hubei(3:1) [18] provinces, suggesting that the risk of infection in females should not be overlooked. The high proportion of students in our study is consistent with that reported in a nationwide study in China [22]. Notably, Cluster I showed a significantly higher proportion of cases in the 0–20 age group (14.44% vs. 4.59% ~ 6.46%) but a lower proportion in the 41–60 age group (37.20% vs. 46.25% ~ 49.45%) compared with other areas. We speculate that the lower proportion in the 41–60 age group may reflect a longer history of HFRS vaccination targeting 16–60 year-olds in this region, given that vaccination is generally accepted as effective in reducing infection risk [13,23]. Conversely, younger individuals—especially those under 16—remained largely unvaccinated and thus faced higher infection risk given the high endemic intensity, although this interpretation lacks direct support from individual-level vaccination data. It is worth noting that the proportion of farmers in all clusters is above 70%, while the proportion in non-cluster areas is less than 60%, suggesting that there are certain differences in the main transmission patterns in different regions.

HFRS cases in Jiangxi Province were predominantly mild or moderate (79.81%), with severe and critical cases accounting for 20.19%. A similar distribution was reported in neighboring Hubei Province [24]—mild and moderate: 80.26%; severe and critical: 19.74%. Notably, marked variation in clinical severity was observed across different clusters. For instance, the proportion of severe cases in Cluster I (22.41%) was significantly higher than that in Cluster II (2.62%). Geographical separation between Cluster I and Cluster II, coupled with the barrier effect of Poyang Lake, likely facilitated habitat differentiation, leading to disparities in the predominant reservoir hosts and major circulating pathogens between the two regions. Laboratory evidence from one county in Cluster I demonstrated the exclusive presence of HTNV and the absence of SEOV [15]. The Cluster II is adjacent to western Zhejiang Province, a region historically endemic for HFRS [25], and the SEOV strains predominantly circulating in Jiangxi and Zhejiang provinces exhibit certain similarities [22]. Moreover, the overall CFR of HFRS in Jiangxi Province(1.79%) was higher than that in Zhejiang Province(0.31% ~ 0.47%) [21,26]. Previous studies [27,28] have indicated that SEOV infection typically leads to milder clinical manifestations than HTNV infection. Based on these ecological and epidemiological clues, it is plausible—though speculative and requiring direct virological confirmation—that cases in Cluster II were primarily infected with milder SEOV strains, whereas Cluster I experienced a higher proportion of HTNV-related severe cases. However, we emphasize that this genotypic attribution is inferred from geographic and literature-based evidence rather than from direct viral sequencing data from our cohort. Additionally, we found that the highest CFR occurred in non-clustered areas. We speculate that this phenomenon may be related to limited diagnostic awareness of HFRS among local primary healthcare providers and treatment delays associated with the need for referral to higher-level hospitals for laboratory confirmation, although this speculation lacks direct support from individual-level clinical data.

In contrast, the clinical severity distribution in Cluster III closely resembled that in Cluster I. This resemblance may be partially related to their geographical proximity, shared primary rodent reservoirs, and consequent infection with identical viral strains. Moreover, Cluster I had a higher proportion of elderly individuals, which may have further increased the population’s risk of severe disease [29]. Nevertheless, given the absence of individual-level vaccination records in our dataset, this interpretation remains conjectural and should be treated as a hypothesis-generating observation rather than a demonstrated causal pathway.

This study found that the median onset-to-visit interval for HFRS cases in Jiangxi Province was 3 days, comparable to reports from Beijing [30] but longer than that documented in Shaanxi [31]. The interval was longest in Cluster II, significantly exceeding that in other regions—a difference consistently observed across gender, age, and occupational subgroups. This might be associated with the highest proportion of mild type cases in Cluster II, and mild cases exhibited the longest onset-to-visit interval. Additionally, this delay could reflect a combination of factors, including limited accessibility to healthcare facilities, underdeveloped transportation infrastructure, and insufficient local promotion of HFRS prevention knowledge. In contrast, Cluster I had a relatively shorter onset-to-visit interval, which may be associated with its high proportion of severe and critical cases. One possible explanation is that, as a historically high-incidence area, residents of Cluster I probably possess greater awareness of HFRS prevention and control, prompting more timely healthcare seeking after symptom onset. However, we cannot rule out the reverse possibility—patients with more severe symptoms may have been compelled to seek care earlier regardless of their awareness level. This phenomenon aligns with previous findings that milder symptoms are associated with lower urgency to seek care and longer delays [32]. The predominance of cases seeking care at Class II hospitals (local county hospitals) in Cluster I also indirectly suggests that these facilities have accumulated stronger experience in HFRS diagnosis and treatment compared with Class II hospitals elsewhere. Conversely, HFRS cases in Cluster II may not be promptly diagnosed at local Class II hospitals and instead require referral to higher-level facilities for confirmation—a pathway that may partly explain the significantly longer onset-to-visit interval observed in this cluster. Consistently, Cluster II exhibited a substantially lower rate of laboratory confirmation relative to Cluster I (34.96% vs. 69.79%), which further substantiates the above inference. No significant differences in disease severity were observed across occupational groups, but there was a clear occupational differentiation in the choice of hospital level. Homemakers/unemployed individuals and those in other occupations (e.g., self‑employed or freelancers) were more likely to seek care at high‑level hospitals (Class III/III+) compared with farmers, which may be related to their relatively higher socioeconomic status. In contrast, students did not differ significantly from farmers in hospital level choice, which may be associated with the fact that students were predominantly concentrated in Cluster 1, where most cases were treated at local Class III hospitals.

Our observations partially confirmed our hypothesis; however, several limitations should be acknowledged. First, the approximately 33% exclusion rate may have introduced selection bias. Although age and sex distributions were similar between included and excluded cases, severely ill patients may have been preferentially retained due to more complete records, potentially overestimating the proportion of severe/critical cases. We have therefore emphasized relative comparisons between clusters rather than absolute severity estimates. Second, marked heterogeneity in exclusion rates across clusters (ranging from 15.79% to 57.27%) raises concern for differential selection bias, as clusters with higher exclusion rates disproportionately omitted milder cases, potentially distorting inter-cluster comparisons. Third, we lacked virological genotyping data (e.g., HTNV vs. SEOV) and individual-level vaccination records, rendering our interpretations regarding viral and immunological mechanisms speculative and warranting further investigation. Fourth, in the absence of direct measures of health literacy or healthcare access, we used onset-to-visit interval and hospital level as proxy indicators; these variables are inevitably confounded by regional infrastructure, healthcare accessibility, and health system factors, and we have accordingly tempered our interpretations throughout the manuscript. Finally, despite these efforts, the retrospective nature of our surveillance data—spanning 13 years—precludes any definitive determination of directionality for the observed associations between health-seeking behaviors and clinical severity. Our findings should therefore be viewed as hypothesis-generating rather than causal; however, the observed relative patterns across clusters remain the most robust takeaway from this analysis.

The findings of this retrospective study provide exploratory evidence for regional heterogeneity in HFRS severity and healthcare-seeking patterns across Jiangxi Province. Cluster II, geographically separated from high-incidence areas by natural barriers, exhibited lower severe case proportions and longer onset-to-visit intervals, underscoring the need for geographically differentiated public health strategies. Specifically, in high-severity clusters (Cluster I and III), strengthening early warning systems and critical care capacity at local Class II hospitals may be prioritized. In mild-predominant clusters (Cluster II), interventions to shorten onset-to-visit intervals—such as community health education, improved transportation links, or enhanced primary care referral pathways—could be considered, though the relative contribution of each factor requires further investigation. At the provincial level, the observed heterogeneity highlights the importance of geographically stratified surveillance systems to enable timely detection of emerging high-risk areas and adaptive resource allocation. Importantly, these policy considerations are hypothesis-generating and derived from observational associations, not from demonstrated causal mechanisms. Future prospective studies incorporating viral genotyping, health literacy assessments, and healthcare access metrics are warranted to validate these observations and refine targeted interventions.

## Supporting information

S1 Text The counties and districts included in different clusters. (DOCX)

S2 Text The diagnostic criteria of HFRS cases. (DOCX)

S3 Text The information of epidemiological investigations. (DOCX)

S4 Text The clinical types of HFRS cases. (DOCX)

S1 Data The underlying numerical data for Fig 3. (PDF)

S2 Data The underlying numerical data for Fig 4. (PDF)

S3 Data The underlying numerical data for Fig 5. (PDF)

S1 Table The distribution of clinical severity for cases by clusters. (DOCX)

S2 Table The distribution of hospital levels by clusters. (DOCX)

S1 Fig Comparison of reported and analysis cases. (TIF)

S2 Fig Comparison of excluded and included cases. (TIF)

S3 Fig Trends in reported incidence rates by clusters. (TIF)

S4 Fig Comparison of the onset-to-visit interval by clusters. ***: p.adj < 0.001.
(TIF)

S5 Fig Comparison of the onset-to-visit interval across clusters. (A) Stratified by gender; (B) Stratified by age; (C) Stratified by occupation. *: p.adj < 0.05; **: p.adj < 0.01; ***: p.adj < 0.001.
(TIF)

S6 Fig Comparison of the onset-to-visit interval by clinical types. ***: p.adj < 0.001.
(TIF)

S7 Fig Comparison of the onset-to-visit interval by hospital level. ***: p.adj < 0.001.
(TIF)

S8 Fig Distribution of hospital levels by clusters. (TIF)

S9 Fig Distribution of hospital levels by clinical types. (TIF)

S10 Fig Comparison of hospital level distribution by clinical types and clusters. Values below 1% were not labeled.
(TIF)
