# Supplementary material for: Clinical severity and health-seeking behaviors of cases with hemorrhagic fever with renal syndrome: A comparative study across spatial clusters in Jiangxi Province, China
Source: PLoS Negl Trop Dis. 2026 Sep 23;20(9):e0014746. doi: 10.1371/journal.pntd.0014746 (PMC13626467; doi:10.1371/journal.pntd.0014746)
Supplement: Supplementary file 1 [file pntd.0014746.s005.pdf]

Year	Cluster category	Onset-to-visit interval	Count	Total	Proportion(%)
**2011**	Cluster I	0–7	172	200	86.00
**2011**	Cluster I	8–14	22	200	11.00
**2011**	Cluster I	15–30	5	200	2.50
**2011**	Cluster I	31–60	1	200	0.50
**2011**	Cluster II	0–7	72	108	66.67
**2011**	Cluster II	8–14	19	108	17.59
**2011**	Cluster II	15–30	13	108	12.04
**2011**	Cluster II	31–60	4	108	3.70
**2011**	Cluster III	0–7	35	43	81.40
**2011**	Cluster III	8–14	5	43	11.63
**2011**	Cluster III	15–30	2	43	4.65
**2011**	Cluster III	31–60	1	43	2.33
**2011**	Non-cluster area	0–7	35	49	71.43
**2011**	Non-cluster area	8–14	8	49	16.33
**2011**	Non-cluster area	15–30	3	49	6.12
**2011**	Non-cluster area	31–60	3	49	6.12
**2012**	Cluster I	0–7	200	207	96.62
**2012**	Cluster I	8–14	6	207	2.90
**2012**	Cluster I	15–30	1	207	0.48
**2012**	Cluster II	0–7	83	121	68.60
**2012**	Cluster II	8–14	28	121	23.14
**2012**	Cluster II	15–30	9	121	7.44
**2012**	Cluster II	31–60	1	121	0.83
**2012**	Cluster III	0–7	61	64	95.31
**2012**	Cluster III	8–14	3	64	4.69
**2012**	Non-cluster area	0–7	20	30	66.67
**2012**	Non-cluster area	8–14	9	30	30.00
**2012**	Non-cluster area	15–30	1	30	3.33
**2013**	Cluster I	0–7	274	280	97.86
**2013**	Cluster I	8–14	3	280	1.07
**2013**	Cluster I	15–30	3	280	1.07
**2013**	Cluster II	0–7	66	113	58.41
**2013**	Cluster II	8–14	31	113	27.43
**2013**	Cluster II	15–30	13	113	11.50
**2013**	Cluster II	31–60	3	113	2.65
**2013**	Cluster III	0–7	36	42	85.71
**2013**	Cluster III	8–14	2	42	4.76
**2013**	Cluster III	15–30	4	42	9.52
**2013**	Non-cluster area	0–7	28	37	75.68
**2013**	Non-cluster area	8–14	8	37	21.62
**2013**	Non-cluster area	15–30	1	37	2.70
**2014**	Cluster I	0–7	196	213	92.02
**2014**	Cluster I	8–14	14	213	6.57
**2014**	Cluster I	15–30	3	213	1.41
**2014**	Cluster II	0–7	45	81	55.56
**2014**	Cluster II	8–14	27	81	33.33
**2014**	Cluster II	15–30	7	81	8.64

**2014**	Cluster II	31–60	2	81	2.47
**2014**	Cluster III	0–7	19	23	82.61
**2014**	Cluster III	8–14	3	23	13.04
**2014**	Cluster III	15–30	1	23	4.35
**2014**	Non-cluster area	0–7	30	44	68.18
**2014**	Non-cluster area	8–14	8	44	18.18
**2014**	Non-cluster area	15–30	4	44	9.09
**2014**	Non-cluster area	31–60	2	44	4.55
**2015**	Cluster I	0–7	295	310	95.16
**2015**	Cluster I	8–14	11	310	3.55
**2015**	Cluster I	15–30	4	310	1.29
**2015**	Cluster II	0–7	61	107	57.01
**2015**	Cluster II	8–14	31	107	28.97
**2015**	Cluster II	15–30	14	107	13.08
**2015**	Cluster II	31–60	1	107	0.93
**2015**	Cluster III	0–7	60	65	92.31
**2015**	Cluster III	8–14	2	65	3.08
**2015**	Cluster III	15–30	3	65	4.62
**2015**	Non-cluster area	0–7	67	86	77.91
**2015**	Non-cluster area	8–14	13	86	15.12
**2015**	Non-cluster area	15–30	5	86	5.81
**2015**	Non-cluster area	31–60	1	86	1.16
**2016**	Cluster I	0–7	236	249	94.78
**2016**	Cluster I	8–14	9	249	3.61
**2016**	Cluster I	15–30	3	249	1.20
**2016**	Cluster I	31–60	1	249	0.40
**2016**	Cluster II	0–7	45	79	56.96
**2016**	Cluster II	8–14	16	79	20.25
**2016**	Cluster II	15–30	16	79	20.25
**2016**	Cluster II	31–60	2	79	2.53
**2016**	Cluster III	0–7	41	47	87.23
**2016**	Cluster III	8–14	6	47	12.77
**2016**	Non-cluster area	0–7	49	56	87.50
**2016**	Non-cluster area	8–14	4	56	7.14
**2016**	Non-cluster area	15–30	3	56	5.36
**2017**	Cluster I	0–7	220	236	93.22
**2017**	Cluster I	8–14	10	236	4.24
**2017**	Cluster I	15–30	6	236	2.54
**2017**	Cluster II	0–7	34	53	64.15
**2017**	Cluster II	8–14	8	53	15.09
**2017**	Cluster II	15–30	7	53	13.21
**2017**	Cluster II	31–60	4	53	7.55
**2017**	Cluster III	0–7	27	29	93.10
**2017**	Cluster III	8–14	2	29	6.90
**2017**	Non-cluster area	0–7	27	36	75.00
**2017**	Non-cluster area	8–14	7	36	19.44
**2017**	Non-cluster area	15–30	2	36	5.56
**2018**	Cluster I	0–7	173	181	95.58

**2018**	Cluster I	8–14	7	181	3.87
**2018**	Cluster I	15–30	1	181	0.55
**2018**	Cluster II	0–7	38	76	50.00
**2018**	Cluster II	8–14	15	76	19.74
**2018**	Cluster II	15–30	10	76	13.16
**2018**	Cluster II	31–60	13	76	17.11
**2018**	Cluster III	0–7	41	57	71.93
**2018**	Cluster III	8–14	11	57	19.30
**2018**	Cluster III	15–30	4	57	7.02
**2018**	Cluster III	31–60	1	57	1.75
**2018**	Non-cluster area	0–7	50	57	87.72
**2018**	Non-cluster area	8–14	5	57	8.77
**2018**	Non-cluster area	15–30	1	57	1.75
**2018**	Non-cluster area	31–60	1	57	1.75
**2019**	Cluster I	0–7	103	105	98.10
**2019**	Cluster I	8–14	1	105	0.95
**2019**	Cluster I	15–30	1	105	0.95
**2019**	Cluster II	0–7	68	118	57.63
**2019**	Cluster II	8–14	39	118	33.05
**2019**	Cluster II	15–30	9	118	7.63
**2019**	Cluster II	31–60	2	118	1.69
**2019**	Cluster III	0–7	46	51	90.20
**2019**	Cluster III	8–14	3	51	5.88
**2019**	Cluster III	15–30	2	51	3.92
**2019**	Non-cluster area	0–7	41	57	71.93
**2019**	Non-cluster area	8–14	12	57	21.05
**2019**	Non-cluster area	15–30	4	57	7.02
**2020**	Cluster I	0–7	121	130	93.08
**2020**	Cluster I	8–14	5	130	3.85
**2020**	Cluster I	15–30	3	130	2.31
**2020**	Cluster I	31–60	1	130	0.77
**2020**	Cluster II	0–7	69	91	75.82
**2020**	Cluster II	8–14	8	91	8.79
**2020**	Cluster II	15–30	7	91	7.69
**2020**	Cluster II	31–60	7	91	7.69
**2020**	Cluster III	0–7	34	37	91.89
**2020**	Cluster III	8–14	1	37	2.70
**2020**	Cluster III	15–30	2	37	5.41
**2020**	Non-cluster area	0–7	48	59	81.36
**2020**	Non-cluster area	8–14	8	59	13.56
**2020**	Non-cluster area	15–30	2	59	3.39
**2020**	Non-cluster area	31–60	1	59	1.69
**2021**	Cluster I	0–7	68	77	88.31
**2021**	Cluster I	8–14	5	77	6.49
**2021**	Cluster I	15–30	2	77	2.60
**2021**	Cluster I	31–60	2	77	2.60
**2021**	Cluster II	0–7	49	61	80.33
**2021**	Cluster II	8–14	7	61	11.48

**2021**	Cluster II	15–30	4	61	6.56
**2021**	Cluster II	31–60	1	61	1.64
**2021**	Cluster III	0–7	40	42	95.24
**2021**	Cluster III	8–14	2	42	4.76
**2021**	Non-cluster area	0–7	33	52	63.46
**2021**	Non-cluster area	8–14	11	52	21.15
**2021**	Non-cluster area	15–30	7	52	13.46
**2021**	Non-cluster area	31–60	1	52	1.92
**2022**	Cluster I	0–7	40	42	95.24
**2022**	Cluster I	15–30	2	42	4.76
**2022**	Cluster II	0–7	31	46	67.39
**2022**	Cluster II	8–14	12	46	26.09
**2022**	Cluster II	15–30	3	46	6.52
**2022**	Cluster III	0–7	11	11	100.00
**2022**	Non-cluster area	0–7	15	24	62.50
**2022**	Non-cluster area	8–14	7	24	29.17
**2022**	Non-cluster area	15–30	2	24	8.33
**2023**	Cluster I	0–7	78	89	87.64
**2023**	Cluster I	8–14	9	89	10.11
**2023**	Cluster I	15–30	2	89	2.25
**2023**	Cluster II	0–7	36	50	72.00
**2023**	Cluster II	8–14	11	50	22.00
**2023**	Cluster II	15–30	2	50	4.00
**2023**	Cluster II	31–60	1	50	2.00
**2023**	Cluster III	0–7	27	31	87.10
**2023**	Cluster III	8–14	3	31	9.68
**2023**	Cluster III	15–30	1	31	3.23
**2023**	Non-cluster area	0–7	30	40	75.00
**2023**	Non-cluster area	8–14	4	40	10.00
**2023**	Non-cluster area	15–30	6	40	15.00

The underlying numerical data for Figure 3
