# Supplementary material for: Clinical severity and health-seeking behaviors of cases with hemorrhagic fever with renal syndrome: A comparative study across spatial clusters in Jiangxi Province, China
Source: PLoS Negl Trop Dis. 2026 Sep 23;20(9):e0014746. doi: 10.1371/journal.pntd.0014746 (PMC13626467; doi:10.1371/journal.pntd.0014746)
Supplement: Supplementary file 2 [file pntd.0014746.s006.pdf]

Year	Cluster category	Hospital level	Count	Total	Proportion(%)
**2011**	Cluster I	Class I	4	197	2.03
**2011**	Cluster I	Class II	96	197	48.73
**2011**	Cluster I	Class III	80	197	40.61
**2011**	Cluster I	Class III+	17	197	8.63
**2011**	Cluster II	Class I	6	109	5.50
**2011**	Cluster II	Class II	74	109	67.89
**2011**	Cluster II	Class III	29	109	26.61
**2011**	Cluster III	Class I	2	42	4.76
**2011**	Cluster III	Class II	18	42	42.86
**2011**	Cluster III	Class III	21	42	50.00
**2011**	Cluster III	Class III+	1	42	2.38
**2011**	Non-cluster area	Class II	10	48	20.83
**2011**	Non-cluster area	Class III	21	48	43.75
**2011**	Non-cluster area	Class III+	17	48	35.42
**2012**	Cluster I	Class II	132	204	64.71
**2012**	Cluster I	Class III	50	204	24.51
**2012**	Cluster I	Class III+	22	204	10.78
**2012**	Cluster II	Class I	5	121	4.13
**2012**	Cluster II	Class II	73	121	60.33
**2012**	Cluster II	Class III	43	121	35.54
**2012**	Cluster III	Class II	12	64	18.75
**2012**	Cluster III	Class III	46	64	71.88
**2012**	Cluster III	Class III+	6	64	9.38
**2012**	Non-cluster area	Class II	8	29	27.59
**2012**	Non-cluster area	Class III	9	29	31.03
**2012**	Non-cluster area	Class III+	12	29	41.38
**2013**	Cluster I	Class II	184	277	66.43
**2013**	Cluster I	Class III	70	277	25.27
**2013**	Cluster I	Class III+	23	277	8.30
**2013**	Cluster II	Class I	1	113	0.88
**2013**	Cluster II	Class II	73	113	64.60
**2013**	Cluster II	Class III	38	113	33.63
**2013**	Cluster II	Class III+	1	113	0.88
**2013**	Cluster III	Class II	3	42	7.14
**2013**	Cluster III	Class III	33	42	78.57
**2013**	Cluster III	Class III+	6	42	14.29
**2013**	Non-cluster area	Class II	18	37	48.65
**2013**	Non-cluster area	Class III	6	37	16.22
**2013**	Non-cluster area	Class III+	13	37	35.14
**2014**	Cluster I	Class II	139	212	65.57
**2014**	Cluster I	Class III	44	212	20.75
**2014**	Cluster I	Class III+	29	212	13.68
**2014**	Cluster II	Class II	47	82	57.32
**2014**	Cluster II	Class III	35	82	42.68
**2014**	Cluster III	Class II	3	23	13.04
**2014**	Cluster III	Class III	20	23	86.96
**2014**	Non-cluster area	Class II	13	44	29.55

**2014**	Non-cluster area	Class III	14	44	31.82
**2014**	Non-cluster area	Class III+	17	44	38.64
**2015**	Cluster I	Class I	3	286	1.05
**2015**	Cluster I	Class II	194	286	67.83
**2015**	Cluster I	Class III	45	286	15.73
**2015**	Cluster I	Class III+	44	286	15.38
**2015**	Cluster II	Class I	1	107	0.93
**2015**	Cluster II	Class II	65	107	60.75
**2015**	Cluster II	Class III	40	107	37.38
**2015**	Cluster II	Class III+	1	107	0.93
**2015**	Cluster III	Class II	18	64	28.13
**2015**	Cluster III	Class III	29	64	45.31
**2015**	Cluster III	Class III+	17	64	26.56
**2015**	Non-cluster area	Class II	19	83	22.89
**2015**	Non-cluster area	Class III	17	83	20.48
**2015**	Non-cluster area	Class III+	47	83	56.63
**2016**	Cluster I	Class II	143	232	61.64
**2016**	Cluster I	Class III	57	232	24.57
**2016**	Cluster I	Class III+	32	232	13.79
**2016**	Cluster II	Class I	1	82	1.22
**2016**	Cluster II	Class II	41	82	50.00
**2016**	Cluster II	Class III	40	82	48.78
**2016**	Cluster III	Class II	3	47	6.38
**2016**	Cluster III	Class III	30	47	63.83
**2016**	Cluster III	Class III+	14	47	29.79
**2016**	Non-cluster area	Class II	24	56	42.86
**2016**	Non-cluster area	Class III	15	56	26.79
**2016**	Non-cluster area	Class III+	17	56	30.36
**2017**	Cluster I	Class II	130	224	58.04
**2017**	Cluster I	Class III	53	224	23.66
**2017**	Cluster I	Class III+	41	224	18.30
**2017**	Cluster II	Class II	35	54	64.81
**2017**	Cluster II	Class III	18	54	33.33
**2017**	Cluster II	Class III+	1	54	1.85
**2017**	Cluster III	Class II	3	29	10.34
**2017**	Cluster III	Class III	15	29	51.72
**2017**	Cluster III	Class III+	11	29	37.93
**2017**	Non-cluster area	Class II	7	37	18.92
**2017**	Non-cluster area	Class III	13	37	35.14
**2017**	Non-cluster area	Class III+	17	37	45.95
**2018**	Cluster I	Class II	143	181	79.01
**2018**	Cluster I	Class III	6	181	3.31
**2018**	Cluster I	Class III+	32	181	17.68
**2018**	Cluster II	Class II	34	81	41.98
**2018**	Cluster II	Class III	47	81	58.02
**2018**	Cluster III	Class II	14	57	24.56
**2018**	Cluster III	Class III	13	57	22.81
**2018**	Cluster III	Class III+	30	57	52.63

**2018**	Non-cluster area	Class I	1	57	1.75
**2018**	Non-cluster area	Class II	14	57	24.56
**2018**	Non-cluster area	Class III	27	57	47.37
**2018**	Non-cluster area	Class III+	15	57	26.32
**2019**	Cluster I	Class II	93	105	88.57
**2019**	Cluster I	Class III	1	105	0.95
**2019**	Cluster I	Class III+	11	105	10.48
**2019**	Cluster II	Class II	38	119	31.93
**2019**	Cluster II	Class III	74	119	62.18
**2019**	Cluster II	Class III+	7	119	5.88
**2019**	Cluster III	Class II	16	50	32.00
**2019**	Cluster III	Class III	18	50	36.00
**2019**	Cluster III	Class III+	16	50	32.00
**2019**	Non-cluster area	Class I	1	58	1.72
**2019**	Non-cluster area	Class II	13	58	22.41
**2019**	Non-cluster area	Class III	22	58	37.93
**2019**	Non-cluster area	Class III+	22	58	37.93
**2020**	Cluster I	Class II	99	128	77.34
**2020**	Cluster I	Class III	1	128	0.78
**2020**	Cluster I	Class III+	28	128	21.88
**2020**	Cluster II	Class II	28	94	29.79
**2020**	Cluster II	Class III	57	94	60.64
**2020**	Cluster II	Class III+	9	94	9.57
**2020**	Cluster III	Class II	7	35	20.00
**2020**	Cluster III	Class III	7	35	20.00
**2020**	Cluster III	Class III+	21	35	60.00
**2020**	Non-cluster area	Class II	17	59	28.81
**2020**	Non-cluster area	Class III	21	59	35.59
**2020**	Non-cluster area	Class III+	21	59	35.59
**2021**	Cluster I	Class II	53	77	68.83
**2021**	Cluster I	Class III	3	77	3.90
**2021**	Cluster I	Class III+	21	77	27.27
**2021**	Cluster II	Class II	16	62	25.81
**2021**	Cluster II	Class III	39	62	62.90
**2021**	Cluster II	Class III+	7	62	11.29
**2021**	Cluster III	Class II	10	40	25.00
**2021**	Cluster III	Class III	13	40	32.50
**2021**	Cluster III	Class III+	17	40	42.50
**2021**	Non-cluster area	Class II	7	53	13.21
**2021**	Non-cluster area	Class III	26	53	49.06
**2021**	Non-cluster area	Class III+	20	53	37.74
**2022**	Cluster I	Class II	29	42	69.05
**2022**	Cluster I	Class III+	13	42	30.95
**2022**	Cluster II	Class II	10	46	21.74
**2022**	Cluster II	Class III	27	46	58.70
**2022**	Cluster II	Class III+	9	46	19.57
**2022**	Cluster III	Class II	6	11	54.55
**2022**	Cluster III	Class III+	5	11	45.45

**2022**	Non-cluster area	Class II	6	25	24.00
**2022**	Non-cluster area	Class III	6	25	24.00
**2022**	Non-cluster area	Class III+	13	25	52.00
**2023**	Cluster I	Class II	50	89	56.18
**2023**	Cluster I	Class III	22	89	24.72
**2023**	Cluster I	Class III+	17	89	19.10
**2023**	Cluster II	Class II	8	49	16.33
**2023**	Cluster II	Class III	39	49	79.59
**2023**	Cluster II	Class III+	2	49	4.08
**2023**	Cluster III	Class II	17	30	56.67
**2023**	Cluster III	Class III	9	30	30.00
**2023**	Cluster III	Class III+	4	30	13.33
**2023**	Non-cluster area	Class II	15	40	37.50
**2023**	Non-cluster area	Class III	15	40	37.50
**2023**	Non-cluster area	Class III+	10	40	25.00

The underlying numerical data for Figure 4
