# Supplementary material for: Clinical severity and health-seeking behaviors of cases with hemorrhagic fever with renal syndrome: A comparative study across spatial clusters in Jiangxi Province, China
Source: PLoS Negl Trop Dis. 2026 Sep 23;20(9):e0014746. doi: 10.1371/journal.pntd.0014746 (PMC13626467; doi:10.1371/journal.pntd.0014746)
Supplement: Supplementary file 3 [file pntd.0014746.s007.pdf]

Cluster category	Gender	Hospital level	Count	Total	Proportion(%)
**Cluster I**	Female	Class I	2	742	0.27
**Cluster I**	Female	Class II	518	742	69.81
**Cluster I**	Female	Class III	126	742	16.98
**Cluster I**	Female	Class III+	96	742	12.94
**Cluster I**	Male	Class I	5	1512	0.33
**Cluster I**	Male	Class II	967	1512	63.96
**Cluster I**	Male	Class III	306	1512	20.24
**Cluster I**	Male	Class III+	234	1512	15.48
**Cluster II**	Female	Class I	6	365	1.64
**Cluster II**	Female	Class II	177	365	48.49
**Cluster II**	Female	Class III	164	365	44.93
**Cluster II**	Female	Class III+	18	365	4.93
**Cluster II**	Male	Class I	8	754	1.06
**Cluster II**	Male	Class II	365	754	48.41
**Cluster II**	Male	Class III	362	754	48.01
**Cluster II**	Male	Class III+	19	754	2.52
**Cluster III**	Female	Class I	0	153	0.00
**Cluster III**	Female	Class II	35	153	22.88
**Cluster III**	Female	Class III	80	153	52.29
**Cluster III**	Female	Class III+	38	153	24.84
**Cluster III**	Male	Class I	2	381	0.52
**Cluster III**	Male	Class II	95	381	24.93
**Cluster III**	Male	Class III	174	381	45.67
**Cluster III**	Male	Class III+	110	381	28.87
**Non-clustered**	Female	Class I	1	199	0.50
**Non-clustered**	Female	Class II	60	199	30.15
**Non-clustered**	Female	Class III	62	199	31.16
**Non-clustered**	Female	Class III+	76	199	38.19
**Non-clustered**	Male	Class I	1	427	0.23
**Non-clustered**	Male	Class II	111	427	26.00
**Non-clustered**	Male	Class III	150	427	35.13
**Non-clustered**	Male	Class III+	165	427	38.64

The underlying numerical data for Figure 5(A)

Cluster category	Age group	Hospital level	Count	Total	Proportion(%)
Cluster I	0–15	Class I	0	244	0.00
Cluster I	0–15	Class II	135	244	55.33
Cluster I	0–15	Class III	72	244	29.51
Cluster I	0–15	Class III+	37	244	15.16
Cluster I	16–60	Class I	6	1436	0.42
Cluster I	16–60	Class II	936	1436	65.18
Cluster I	16–60	Class III	286	1436	19.92
Cluster I	16–60	Class III+	208	1436	14.48
Cluster I	≥61	Class I	1	574	0.17
Cluster I	≥61	Class II	414	574	72.13
Cluster I	≥61	Class III	74	574	12.89
Cluster I	≥61	Class III+	85	574	14.81
Cluster II	0–15	Class I	0	37	0.00
Cluster II	0–15	Class II	14	37	37.84
Cluster II	0–15	Class III	18	37	48.65
Cluster II	0–15	Class III+	5	37	13.51
Cluster II	16–60	Class I	8	785	1.02
Cluster II	16–60	Class II	378	785	48.15
Cluster II	16–60	Class III	371	785	47.26
Cluster II	16–60	Class III+	28	785	3.57
Cluster II	≥61	Class I	6	297	2.02
Cluster II	≥61	Class II	150	297	50.51
Cluster II	≥61	Class III	137	297	46.13
Cluster II	≥61	Class III+	4	297	1.35
Cluster III	0–15	Class I	0	26	0.00
Cluster III	0–15	Class II	2	26	7.69
Cluster III	0–15	Class III	14	26	53.85
Cluster III	0–15	Class III+	10	26	38.46
Cluster III	16–60	Class I	2	396	0.51
Cluster III	16–60	Class II	95	396	23.99
Cluster III	16–60	Class III	187	396	47.22
Cluster III	16–60	Class III+	112	396	28.28
Cluster III	≥61	Class I	0	112	0.00
Cluster III	≥61	Class II	33	112	29.46
Cluster III	≥61	Class III	53	112	47.32
Cluster III	≥61	Class III+	26	112	23.21
Non-clustered	0–15	Class I	0	16	0.00
Non-clustered	0–15	Class II	2	16	12.50
Non-clustered	0–15	Class III	8	16	50.00
Non-clustered	0–15	Class III+	6	16	37.50
Non-clustered	16–60	Class I	2	461	0.43
Non-clustered	16–60	Class II	117	461	25.38
Non-clustered	16–60	Class III	158	461	34.27
Non-clustered	16–60	Class III+	184	461	39.91
Non-clustered	≥61	Class I	0	149	0.00
Non-clustered	≥61	Class II	52	149	34.90
Non-clustered	≥61	Class III	46	149	30.87

Non-clustered	≥61	Class III+	51	149	34.23

The underlying numerical data for Figure 5(B)

Cluster category	Occupation	Hospital level	Count	Total	Proportion(%)
Cluster I	Farmers	Class I	4	1593	0.25
Cluster I	Farmers	Class II	1109	1593	69.62
Cluster I	Farmers	Class III	310	1593	19.46
Cluster I	Farmers	Class III+	170	1593	10.67
Cluster I	Housework	Class I	0	110	0.00
Cluster I	Housework	Class II	54	110	49.09
Cluster I	Housework	Class III	7	110	6.36
Cluster I	Housework	Class III+	49	110	44.55
Cluster I	Students	Class I	0	240	0.00
Cluster I	Students	Class II	139	240	57.92
Cluster I	Students	Class III	66	240	27.50
Cluster I	Students	Class III+	35	240	14.58
Cluster I	Workers	Class I	1	79	1.27
Cluster I	Workers	Class II	62	79	78.48
Cluster I	Workers	Class III	11	79	13.92
Cluster I	Workers	Class III+	5	79	6.33
Cluster I	Others	Class I	2	232	0.86
Cluster I	Others	Class II	121	232	52.16
Cluster I	Others	Class III	38	232	16.38
Cluster I	Others	Class III+	71	232	30.60
Cluster II	Farmers	Class I	12	841	1.43
Cluster II	Farmers	Class II	430	841	51.13
Cluster II	Farmers	Class III	380	841	45.18
Cluster II	Farmers	Class III+	19	841	2.26
Cluster II	Housework	Class I	0	89	0.00
Cluster II	Housework	Class II	38	89	42.70
Cluster II	Housework	Class III	43	89	48.31
Cluster II	Housework	Class III+	8	89	8.99
Cluster II	Students	Class I	0	44	0.00
Cluster II	Students	Class II	18	44	40.91
Cluster II	Students	Class III	20	44	45.45
Cluster II	Students	Class III+	6	44	13.64
Cluster II	Workers	Class I	1	19	5.26
Cluster II	Workers	Class II	7	19	36.84
Cluster II	Workers	Class III	11	19	57.89
Cluster II	Workers	Class III+	0	19	0.00
Cluster II	Others	Class I	1	126	0.79
Cluster II	Others	Class II	49	126	38.89
Cluster II	Others	Class III	72	126	57.14
Cluster II	Others	Class III+	4	126	3.17
Cluster III	Farmers	Class I	2	412	0.49
Cluster III	Farmers	Class II	105	412	25.49
Cluster III	Farmers	Class III	207	412	50.24
Cluster III	Farmers	Class III+	98	412	23.79
Cluster III	Housework	Class I	0	28	0.00
Cluster III	Housework	Class II	9	28	32.14
Cluster III	Housework	Class III	6	28	21.43

Cluster III	Housework	Class III+	13	28	46.43
Cluster III	Students	Class I	0	27	0.00
Cluster III	Students	Class II	2	27	7.41
Cluster III	Students	Class III	16	27	59.26
Cluster III	Students	Class III+	9	27	33.33
Cluster III	Workers	Class I	0	16	0.00
Cluster III	Workers	Class II	1	16	6.25
Cluster III	Workers	Class III	11	16	68.75
Cluster III	Workers	Class III+	4	16	25.00
Cluster III	Others	Class I	0	51	0.00
Cluster III	Others	Class II	13	51	25.49
Cluster III	Others	Class III	14	51	27.45
Cluster III	Others	Class III+	24	51	47.06
Non-clustered	Farmers	Class I	2	376	0.53
Non-clustered	Farmers	Class II	133	376	35.37
Non-clustered	Farmers	Class III	131	376	34.84
Non-clustered	Farmers	Class III+	110	376	29.26
Non-clustered	Housework	Class I	0	75	0.00
Non-clustered	Housework	Class II	11	75	14.67
Non-clustered	Housework	Class III	18	75	24.00
Non-clustered	Housework	Class III+	46	75	61.33
Non-clustered	Students	Class I	0	23	0.00
Non-clustered	Students	Class II	5	23	21.74
Non-clustered	Students	Class III	10	23	43.48
Non-clustered	Students	Class III+	8	23	34.78
Non-clustered	Workers	Class I	0	36	0.00
Non-clustered	Workers	Class II	6	36	16.67
Non-clustered	Workers	Class III	10	36	27.78
Non-clustered	Workers	Class III+	20	36	55.56
Non-clustered	Others	Class I	0	116	0.00
Non-clustered	Others	Class II	16	116	13.79
Non-clustered	Others	Class III	43	116	37.07
Non-clustered	Others	Class III+	57	116	49.14

The underlying numerical data for Figure 5(C)
