# Supplementary material for: Clinical severity and health-seeking behaviors of cases with hemorrhagic fever with renal syndrome: A comparative study across spatial clusters in Jiangxi Province, China
Source: PLoS Negl Trop Dis. 2026 Sep 23;20(9):e0014746. doi: 10.1371/journal.pntd.0014746 (PMC13626467; doi:10.1371/journal.pntd.0014746)
Supplement: Supplementary file 4 [file pntd.0014746.s001.docx]

**S1 Text. The counties and districts included in different clusters**

The cluster category of the counties is based on previous study[1]. The counties of Cluster I: Wuning, Jingan, Anyi, Xinjian, Fengxin, Yifeng, Shanggao, Gaoan.The counties of Cluster II:Yushan, Guangxin, Xinzhou, Guangfeng, Hengfeng, Yanshan. The counties of Cluster III:Fengcheng, Linchuan, Jinxi, Zixi, Lichuan, Nancheng, Nanfeng, Guangchang, Yihuang, Chongren, Le’an, Yongfeng, Xingan.

**References**

[1].	Yang S, Gao Y, Liu X, Liu X, Liu Y, Metelmann S, et al. Spatiotemporal dynamics of	hemorrhagic fever with renal syndrome in Jiangxi province, China. Sci Rep. 2020;10: 14291.	doi:10.1038/s41598-020-70761-0
