# Supplementary material for: Clinical severity and health-seeking behaviors of cases with hemorrhagic fever with renal syndrome: A comparative study across spatial clusters in Jiangxi Province, China
Source: PLoS Negl Trop Dis. 2026 Sep 23;20(9):e0014746. doi: 10.1371/journal.pntd.0014746 (PMC13626467; doi:10.1371/journal.pntd.0014746)
Supplement: Supplementary file 5 [file pntd.0014746.s002.docx]

**S2 Text. The diagnostic criteria of HFRS cases**

HFRS cases were classified as suspected, clinically diagnostic or confirmed cases according to the health industry standard of the People’s Republic of China for diagnostic criteria of HFRS. Briefly, 1) a patient with epidemiological history and clinical manifestations of fever or gastrointestinal symptoms was defined as a suspected case; 2) a suspected case with hypotension, renal function impairment, increased peripheral blood cell counts and thrombocytopenia, and positive urine protein was defined as a clinically diagnosed case; and (3) a clinically diagnosed case with one or more of the following criteria was defined as a confirmed case: positive serum-specific IgM antibody; specific IgG antibody 4 times higher than that in the acute phase; and Hantavirus RNA is positive or Hantavirus is isolated.
