# Supplementary material for: Clinical severity and health-seeking behaviors of cases with hemorrhagic fever with renal syndrome: A comparative study across spatial clusters in Jiangxi Province, China
Source: PLoS Negl Trop Dis. 2026 Sep 23;20(9):e0014746. doi: 10.1371/journal.pntd.0014746 (PMC13626467; doi:10.1371/journal.pntd.0014746)
Supplement: Supplementary file 6 [file pntd.0014746.s003.docx]

**S3 Text. The information of epidemiological investigations**

The information includes general information (e.g. name, gender, age, primary occupation, ethnicity, current address, phone number), clinical severity, healthcare-seeking behaviors (e.g. onset time, place of onset, visiting time, diagnosed time, inpatient hospital). A total of 13 indicators were included in the analysis.
