# Supplementary material for: Clinical severity and health-seeking behaviors of cases with hemorrhagic fever with renal syndrome: A comparative study across spatial clusters in Jiangxi Province, China
Source: PLoS Negl Trop Dis. 2026 Sep 23;20(9):e0014746. doi: 10.1371/journal.pntd.0014746 (PMC13626467; doi:10.1371/journal.pntd.0014746)
Supplement: Supplementary file 7 [file pntd.0014746.s004.docx]

S4 Text. The clinical types of HFRS cases

According to the clinical characteristics of patients, such as body temperature, blood pressure, urine output, edema, and renal injury indicators like urinary protein and urea nitrogen, the severity of HFRS was divided into four clinical types [1,2]. The four clinical types were as follows: (1) the mild group had renal injury without hypotension and oliguria; (2) the moderate group had obvious uremia, bulbar conjunctival edema, skin and mucosal hemorrhage, and acute renal failure with typical oliguria; (3) the severe group showed severe uremia, bulbar conjunctiva and peritoneal or pleural effusion, skin and mucosal bleeding, hypotension, and acute renal failure with oliguria (patients with daily output of 50–500 mL ≤5 days or urine output <100 mL/day ≤2 days); (4) the critically ill group had one or more of the following manifestations compared with the severe group: refractory shock (≥2 days), heart failure, pulmonary edema, visceral hemorrhage, cerebral edema, severe secondary infection, and severe acute renal failure with oliguria (urine volume 50–500 mL/day >5 days) or anuria (urine <100 mL/day >2 days) or blood urea nitrogen (BUN) >42.84 mmol/L.

1.	Bai X, Xu Z. Hemorrhagic fever with renal syndrome. Beijing: People’s Medical Publishing House; 2013.

2.	Yang Z, Hu Q, Feng Z, Sun Y. Development and validation of a nomogram for predicting severity in patients with hemorrhagic fever with renal syndrome: A retrospective study. Open Med (Wars). 2021;16: 944–954. doi:10.1515/med-2021-0307
