# Supplementary material for: Clinical severity and health-seeking behaviors of cases with hemorrhagic fever with renal syndrome: A comparative study across spatial clusters in Jiangxi Province, China
Source: PLoS Negl Trop Dis. 2026 Sep 23;20(9):e0014746. doi: 10.1371/journal.pntd.0014746 (PMC13626467; doi:10.1371/journal.pntd.0014746)
Supplement: Supplementary file 8 [file pntd.0014746.s008.docx]

**S1 Table. The distribution of clinical severity for cases by clusters**

| Clinical type | Cluster I | Cluster II | Cluster III | Non-clustered |
| --- | --- | --- | --- | --- |
| Mild | 794 (34.96%) | 801 (75.00%) | 157 (34.20%) | 412 (68.10%) |
| Moderate | 817 (35.98%) | 225 (21.07%) | 201 (43.79%) | 107 (17.69%) |
| Severe | 509 (22.41%) | 28 (2.62%) | 80 (17.43%) | 51 (8.43%) |
| Critical | 151 (6.65%) | 14 (1.31%) | 21 (4.58%) | 35 (5.79%) |
