# Supplementary material for: Clinical severity and health-seeking behaviors of cases with hemorrhagic fever with renal syndrome: A comparative study across spatial clusters in Jiangxi Province, China
Source: PLoS Negl Trop Dis. 2026 Sep 23;20(9):e0014746. doi: 10.1371/journal.pntd.0014746 (PMC13626467; doi:10.1371/journal.pntd.0014746)
Supplement: Supplementary file 9 [file pntd.0014746.s009.docx]

**S2 Table. The distribution of hospital levels by clusters**

| Hospital level | Cluster I | Cluster II | Cluster III | Non-clustered |
| --- | --- | --- | --- | --- |
| Class I | 7 (0.31%) | 14 (1.25%) | 2 (0.37%) | 2 (0.32%) |
| Class II | 1485 (65.88%) | 542 (48.44%) | 130 (24.34%) | 171 (27.32%) |
| Class III | 432 (19.17%) | 526 (47.01%) | 254 (47.57%) | 212 (33.87%) |
| Class III+ | 330 (14.64%) | 37 (3.31%) | 148 (27.72%) | 241 (38.50%) |
